# Root Filling. No. 1

**Published:** 1867-08

**Authors:** Andrew B. Brookins


(To be Continued.)

---

### ARTICLE II.

### *Root Filling. No.* 1.

By Andrew B. Brookins, D.D.S.

What can I say with reference to the devitalization of dental nerves that has not already been said by those, something my seniors, both in practice and in years? Or what shall we do that has been left undone in the treatment of this class of cases, preparatory to the introduction of gold in the canals and crown cavities?

Among the practitioners of General Medicine, we have the infinitisimal Homœopathy, the heroic Allopathy, and the so-called Eclectic; the latter assuming the assurance of the first, the thunder of the second, with ostensible result of the trio. Let us see if we have not something of this kind in special medicine.

There is a class of operators, not in the minority by any means, that recommend and adopt too, the practice of filling over exposed commissures and nerves in dental caries; of filling crowns, the canals of which still contain vitalized nerves—with concomitant vessels; of filling crowns when the devitalization of these nerves have been effected either through the agency of therapia, the instrument, or by previous excessive inflammatory action, induced by concussion, &c., and peradventure the putrescent mass still remains in the duct, in contiguity with live tissue.

They tell us their patients never return with, or complain of odontalgia or neuralgia. Painful oral or facial abcesses, dental fistula, exfoliations, &c., never make their appearance to break the refreshing slumbers of innocence, to annoy their quiet composure, or mar their beautiful faces. No latent nervous diseases that "human flesh is heir to" are ever developed,—long and weary years of feeble health with shattered nerves and enervated frames.

We will call the advocates of this "system" of practice "conservative," for we must take cognizance of the fact, that there is a vast difference between those who benefit their patients by organizing a *rational* course of treatment, (allopathy) by making them the beneficiaries of valuable professional services.

Let the labor be never so wearisome, your skill and patience be never so provoked, trusting in God and a clear conscience, rendering unto your patients your duty full and nothing wanting. I say there is a moral and a professional distinction, as well between those who benefit their patients by the rendition of actual services and those who withhold that service under the hallucination of speculative medicine, *similia similibus*, &c. We then have conservative as opposed to rational or essential treatment: and the question need not be asked, which shall we embrace, for "plugs" and "gutters" have had their days of inglorious supremacy, and can boast of their "ten thousand" slain.

It is unnecessary to designate between those who adopt this "convervatism" because the easier and fraught with less trouble, and those who are supposed to possess intelligence and christian sympathy, responsive to the tearful sufferings of those they are called upon to relieve.

The result must be always in harmony with the means, and the same end obtains whatever the motives of the operator. No matter if the question is raised in his mind

"what will be the finality if I destroy the life of this dental commissure, remove it and introduce in place a gold filling," leaving the canal filled with a devitalised mass to go through the process of decomposition,—and suppuration—absorption being quite out of question, save a sufficiency to color the organ throughout, adding insult to injury.

I cannot see how intelligent operators need look for other results than the formation of pus, and of course its discharge at a point offering least resistance. Pathologically, I see no distinctive premises between these cases, and what we can readily conceive to occur in general surgery. Take a carious tibia for example: will the surgeon cure this wasting, exfoliating bone by damming up the flowing purulent sanies in its uninterrupted current through the soft parts? Will he bind up the mouths of these fistulas at their openings through the integument, by compress and bandage or adhesesive strips and wait? Waiting for what? for pyemia or hectic fever and wasting attrition of muscle and of life—his experimental "subject" is taken away beyond his "conservative" hand. To bind down and compress the free termini of dental nerves and vessels by the introduction of a filling, let it be the most plastic material that can be manipulated by the careful hand of the operator, it will be sufficiently non-resisting, by acting physically, to effect change of structure and position, physiological functions—inflammation and ultimately devitalization and suppuration follow. I believe it sufficient cause, per se, and I know of no evidence to the contrary, to arouse a morbid diathesis in other and distant parts of the economy, some of the uterine diseases, enervations, hemicrania or cephalalgia; for the brain, the magnum receptaculum of life, the citadel and resting place of the soul, is in voluble sympathy with all parts of the human economy.

Again the dental commissure may be devitalized and it may extend along the entire canal of the organ, if not already effected by the action of natural causes, before the application for treatment had been made by the patient, the former removed from its resting place in the crown of the organ, and the latter allowed to remain in the canal with impunity—as the doctrine of "conservatism" claims; and build up in place of the solution of continuity with a permanent filling and rest on the grinding approximal or other surfaces as if the work had been skillfully, faithfully and professionally executed, and in ninety-nine cases out of a hundred your patient will return, if she is able, and inform you of the presence of fulness or tension in the vicinity of the apex of the root with more or less pain from an unpleasant throbbing to acute, lancinating neuralgia; which generally begins at night and increases in severity until relieved by the discharge of pus to the great relief of the patient; the change of structure disappearing by subsidence of inflammation.

In a given number of cases, the dental fistula makes its appearance more often, immediately in the vicinity of or directly over the extremity of the diseased root; if in the superior maxilla at a line forming the inferior boundary of the oval vestibule, although not by any means confined to one place.

It sometimes happens that the pus effects an opening through the palatal arch and not unfrequently involves the ossa palati which together with contiguous structures slough away, or remain in a cul de sac in the dome of the arch until the knife of the dentist comes to its relief. It however is better in those cases to remove the filling, and cleanse the dental canal with tepid water every 6 or 8 hours till the tumor discharges itself through the tooth. The subsequent treatment hereafter to be considered, will be the same as for subacute pericementitis.

I was consulted in the month of March last, by an intelligent patient, who had been the subject of this kind of convervative *mal*-treatment at the hands of—a "plugger" during the first year of the civil war. Upon examination, I found a tumor occupying the arch of the palate, which from its size and position, materially interfered with her enunciation. My first inclination was to thrust a lancet through the walls of the tumor which was soft and fluctuating, but upon further examination I found a very imperfect filling in the anterior approximal surface of the left central upper incisor which was removed, and by subequent treatment the abcess was cured, and the canal and the anterior approximal cavity filled.

But this is one of many "conservative" cases I have cured within a few months.

There are other points of exit chosen by the pus than above enumerated, in the nasal cavities—in the chamber of Highmore, through the lips, cheeks, chin, &c.; and other results as well, such as exfoliations of the ossa palati, caries or necrosis of the maxillæ, sloughing of soft parts, &c.

Were I asked if there were cases in which I would devitalize the dental commissure, and fill the crown with a permanent filling, allowing the nerve in the canal to remain in its integrity as though we had it in our power to limit the destructibility of, our therapia; or if devitalized, suffer the whole morbid, putrescent mass to remain in the canal—as some advocate, as a professional doctrine—and introduce a filling over it, or if exposed, allow the commissure to remain, and use a cup, and envelop with a material sufficiently hard to prevent the pressure of the filling—(which had there been much hemorrhage I certainly would never do,) I would reply negatively always, unless I wished to entail upon my innocent patient long and wearisome hours and days of suffering, of pain, begotten by my own hand, unguided, however, by the approval of my better judgment. I have never yet seen, and as long as I live, never expect to see an opportunity in which conservatism in Operative Dentistry, can with impunity be resorted to.

There are dentists who are pleased to treat these cases in this way and send out "statistical" evidences of their success. They like it and recommend it, on the contrary, I *abhor* it and wish it could be prevented by statute.

(To be continued.)

---

### ARTICLE III.

### *Nature and Treatment of Decay of the Teeth.*

